# Linking PRMT5 to breast cancer stem cells: New therapeutic opportunities?

**DOI:** 10.1080/23723556.2018.1441628

**Published:** 2018-03-07

**Authors:** Kelly Chiang, Clare C. Davies

**Affiliations:** Institute of Cancer and Genomic Sciences, College of Medical and Dental Sciences, University of Birmingham, UK

**Keywords:** PRMT5, breast cancer stem cells, epigenetics, arginine methylation, FOXP1

## Abstract

The arginine methyltransferase PRMT5 has been increasingly associated with cancer development. Here we describe our recent findings that PRMT5 is a critical regulator of breast cancer stem cell survival via the epigenetic regulation of FOXP1. Consequently, PRMT5 inhibitors could potentially eradicate cancer stem cells thereby preventing tumour relapse.

Protein post-translational modifications expand the functional diversity of the proteome enabling dynamic modulation of cellular processes, but are often deregulated during cancer pathogenesis. Consequently, targeting enzymes that catalyse and remove protein modifications are attractive strategies enabling rational drug design. Arginine methylation, catalysed by protein arginine methyltransferases (PRMTs), was identified over 45 years ago, however the significance of this modification for oncogenesis and malignant progression is only just becoming apparent.^1^ In particular, expression of PRMT5 correlates with poor prognosis of patients and has consequently been attracting significant attention as a novel drug target. Indeed, pre-clinical PRMT5 inhibitors have recently demonstrated impressive efficacy in mouse models of leukaemia.^2^^,^^3^ Despite this, the mechanisms by which PRMT5 contribute to progression of a specific cancer type, particularly that of carcinomas, is largely unknown.

We have recently extended our understanding into the oncogenic nature of PRMT5 through the study of breast cancer stem cells (BCSCs).^4^ BCSCs are a small population of chemoresistant tumour cells that are considered one of the main contributing factors to patient relapse and metastatic disease dissemination.^5^ Interestingly, BCSCs possess a molecular signature similar to cells that have undergone EMT and upregulate stem cell markers such as Oct4 and Nanog.^5^ Given that PRMT5 is known to promote these stem cell traits,^6^^,^^7^ and that high levels are associated with a poor prognosis for breast cancer patients,^4^ we rationalised that PRMT5 activity could be a major driving force in the maintenance of BCSCs.

In our study, we found that PRMT5 levels were significantly elevated in BCSCs compared to bulk differentiated cells, and depletion of PRMT5 reduced the number, proliferative and self-renewal potential of BCSCs. Conversely, overexpression of PRMT5 and its essential cofactor MEP50 increased stem cell frequency *in vivo,* implying that PRMT5 is crucial for driving function. Importantly, to mimic a patient presenting with disease, we depleted PRMT5 in an established tumour xenograft which led to a substantial 12-fold decrease in stem cell numbers as assessed by limiting dilution. Furthermore, we found that treatment of BCSCs isolated from resected primary tumours with a pre-clinical PRMT5 inhibitor significantly reduced BCSC numbers. Collectively, this demonstrates a functional requirement for PRMT5 for the generation and maintenance of the BCSC population and, more importantly, the potential clinical impact of targeting PRMT5.

PRMT5 is a major epigenetic regulator of gene expression, therefore, to gain mechanistic insight we compared the transcriptome of BCSCs before and after PRMT5 depletion. Interestingly, Wnt/β-catenin genes that had previously been reported as PRMT5 targets in leukaemic stem cells^3^ were not differentially expressed, implying that the mechanisms by which PRMT5 regulates cancer stem cell function in different cell types are distinctive. We identified *FOXP1*, a gene which is associated with cancer stem cell function,^8^ as dependent on PRMT5 for expression. Using ChIP analysis, we were able to demonstrate that PRMT5 directly binds to and epigenetically activates the FOXP1 promoter through the di-methylation of H3R2, which in turns leads to the recruitment of WDR5, a component of the SET1/MLL complex, H3K4 tri-methylation and promoter activation (Fig. 1).^4^^,^^9^ Importantly, depletion of FOXP1 suppressed the ability of PRMT5 to drive BCSC proliferation and self-renewal. These findings were surprising as previous immunohistochemical studies correlate high FOXP1 expression with a better patient outcome, hence FOXP1 has largely been considered a breast tumour suppressor gene. In contrast, our xenograft analysis of FOXP1-depleted MCF7 cells clearly showed a retardation of tumour growth. Collectively, these observations indicate FOXP1 is oncogenic, and that epigenetic regulation of the FOXP1 promoter is one mechanism by which PRMT5 drives BCSC function, promoting tumour initiation and drug resistance (Fig. 1). Hence, drug targeting PRMT5 could be an effective way to diminish BCSC activity.

**Figure 1.**
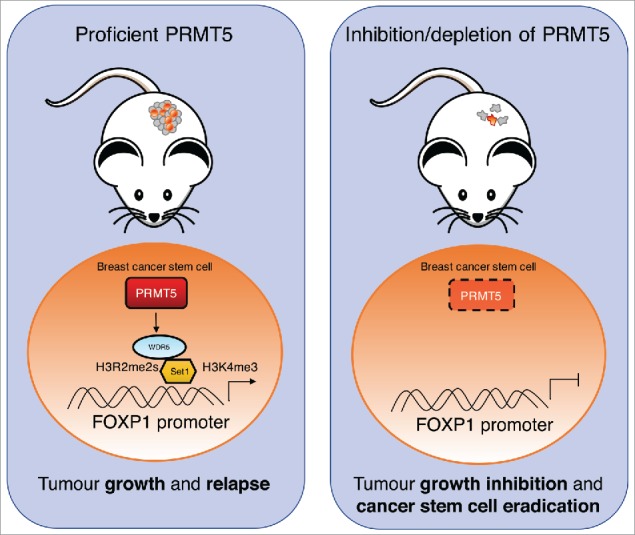
PRMT5 is required for the maintenance and function of breast cancer stem cells. (Left) Breast tumours contain a small population of cells (breast cancer stem cells) that are responsible for tumour growth and relapse (orange cells). These breast cancer stem cells have high levels of PRMT5 which epigenetically regulates the FOXP1 promoter, through H3R2me2s (Histone H3R2 symmetric di-methylation), recruitment of WDR5 and SET1 and subsequent H3K4me3 (Histone H3K4 tri-methylation), resulting in elevated expression of FOXP1. This in turn promotes tumour growth and contributes to the maintenance of cancer stem cells. (Right) Targeting of PRMT5 through either depletion or inhibition with small molecule inhibitors, prevents the activation of FOXP1 expression resulting in slower tumour growth and eradication of the breast cancer stem cell population, thereby highlighting the clinical benefit of targeting PRMT5 in breast cancer.

The success of current therapies for breast cancer remains somewhat marred by poor long-term survival rates due to relapse and metastasis. One major goal for researchers is to prevent relapse by designing therapies to target the hard-to-eliminate cancer stem cells either through eradication of BCSCs or by rendering them more chemosensitive through inducing differentiation. Our study demonstrating that PRMT5 depletion in an established tumour can reduce stem cell frequency is highly significant for the patient, as targeting this enzyme in conjunction with standard chemotherapies, could lead to eradication of all tumours cells, thus preventing relapse. However, further questions remain. We need to fully understand the roles of PRMT5 in this cell type to fully exploit the clinical potential of drug targeting. Although here we describe a role for PRMT5 in the epigenetic regulation of FOXP1, given the pleiotropic roles of this enzyme, it is unlikely to be the only way in which PRMT5 regulates BCSCs. For example, PRMT5 regulates the DNA damage response,^10^ hence this may provide an advantage in protecting against endogenous DNA damage induced by oncogenes, and/or exogenous DNA damage induced by chemotherapies. Another interesting finding is that the mechanisms by which PRMT5 regulates CSCs appear to depend on the cell type, hence highlighting the need for greater understanding. Ultimately, what is the fate of these BCSCs after PRMT5 depletion? Do these cells undergo apoptosis or differentiation, or do they quiesce? These are important questions for directing the design of treatment regimens or long-term patient care.

There is a lot of promise and excitement surrounding epigenetic therapies, and clinical PRMT5 inhibitors have entered phase one trials for leukaemia treatment. Our findings provide compelling evidence that drug targeting PRMT5 could have significant clinical benefit in solid cancers enabling the complete eradication of the tumour-initiating population to improve long-term patient survival.
